# Older adults’ preferences for, adherence to and experiences of two self-management falls prevention home exercise programmes: a comparison between a digital programme and a paper booklet

**DOI:** 10.1186/s12877-020-01592-x

**Published:** 2020-06-15

**Authors:** Linda Mansson, Lillemor Lundin-Olsson, Dawn A. Skelton, Rebecka Janols, Helena Lindgren, Erik Rosendahl, Marlene Sandlund

**Affiliations:** 1 Department of Community Medicine and Rehabilitation, Section of Physiotherapy, Umeå University, Umeå, Sweden; 2 School of Health and Life Sciences, Glasgow Caledonian University, Glasgow, UK; 3 Department of Computing Science, Umeå University, Umeå, Sweden

**Keywords:** Accidental falls, Aged, Aged, 80 and over, Digital health, eHealth, Exercise, Falls prevention, Independent living, mHealth, Self-management

## Abstract

**Background:**

Fall prevention exercise programmes are known to be effective, but access to these programmes is not always possible. The use of eHealth solutions might be a way forward to increase access and reach a wider population. In this feasibility study the aim was to explore the choice of programme, adherence, and self-reported experiences comparing two exercise programmes – a digital programme and a paper booklet.

**Methods:**

A participant preference trial of two self-managed fall prevention exercise interventions. Community-dwelling adults aged 70 years and older exercised independently for four months after one introduction meeting. Baseline information was collected at study start, including a short introduction of the exercise programme, a short physical assessment, and completion of questionnaires. During the four months intervention period, participants self-reported their performed exercises in an exercise diary. At a final meeting, questionnaires about their experiences, and post-assessments, were completed. For adherence analyses data from diaries were used and four subgroups for different levels of participation were compared. Exercise maintenance was followed up with a survey 12 months after study start.

**Results:**

Sixty-seven participants, with mean age 77 ± 4 years were included, 72% were women. Forty-three percent chose the digital programme. Attrition rate was 17% in the digital programme group and 37% in the paper booklet group (*p* = .078). In both groups 50–59% reported exercise at least 75% of the intervention period. The only significant difference for adherence was in the subgroup that completed ≥75% of exercise duration, the digital programme users exercised more minutes per week (*p* = .001). Participants in both groups were content with their programme but digital programme users reported a significantly higher (*p* = .026) degree of being content, and feeling supported by the programme (*p* = .044). At 12 months follow-up 67% of participants using the digital programme continued to exercise regularly compared with 35% for the paper booklet (*p* = .036).

**Conclusions:**

Exercise interventions based on either a digital programme or a paper booklet can be used as a self-managed, independent fall prevention programme. There is a similar adherence in both programmes during a 4-month intervention, but the digital programme seems to facilitate long-term maintenance in regular exercise.

**Trial registration:**

ClinTrial: NCT02916849.

## Background

Falls among the increasing older population is a growing problem in society globally, and actions to prevent falls is necessary. Exercise programmes have been shown to be important interventions for community-dwelling seniors to reduce both rate and risk of falls [1, 2]. Fall and fall related injuries lead to substantial health care costs, for example in 2015 the cost was estimated at $ 50 billion in the US [3]. Not only costs and physical injuries caused by falls have consequences, fear of falling and avoidance of activity may have implications for daily life and social isolation among older adults [4]. Also fear of falling could be reduced by exercise programmes [5].

Various home exercise programmes have shown efficacy in falls prevention. The LiFE study [6] that investigated exercises integrated in daily life, compared with a standard exercise programme, and a control group (gentle exercise) is one example that prevented falls. Another recognised programme is the home-based Otago Exercise Programme, used extensively across the world as an evidence-based falls prevention programme [7–9]. The original programme contains a paper-based booklet with balance and strength exercises and recommends regular walks, together with multiple home visits and phone calls to encourage and motivate participants and to progress the exercises [10]. However, the ProAct65+ trial results show that when the level of motivational support and home visits is less than recommended, the adherence to home-based Otago Exercise Programme (using booklets) is poor [11] and only marginally better with the provision of a DVD with instructions [12]. In the UK, many commissioned services do not provide home visit or telephone support with the same frequency, but hand out the booklets presuming participants continue with the exercises and progress over time [13]. The static nature of paper-based exercise programmes increases the risk that the training routine will become dull and repetitive for the user. Evidence implies that there is a problem with uptake and adherence to exercise in the home setting [14].

According to a review by Sherrington et al. [15] fall prevention interventions that challenge balance and have a higher dose have larger effects. Therefore, adherence to the programme is important as regular practice is most effective [16]. In a systematic review of older adults’ adherence to technology-based exercise programmes, the majority commercially available gaming technology (exergaming = videogames providing physical and/or cognitive exercise) and supervised interventions showed high adherence [17]. Fall prevention exercise interventions using e-Health alternatives, with motivation and support tools, are being studied to discover if such fall preventive interventions can increase adherence. ActiveLifestyle [18, 19] evaluated two different versions of a tablet-based exercise application, one with extra social features, compared to a brochure-based programme over 12 weeks. The study showed higher adherence for the application with social features and both application programmes were used more than the paper programme. An ongoing study, Standing Tall [20] delivers a home-based exercise programme through a tablet and provide additional equipment for practice at home, which is compared with a control group with information only.

Our research group have, in collaboration with older adults, developed an application for a smartphone, tablet or computer which will guide the user to independently perform fall prevention exercises in their own environment [21]. The application Safe Step, a self-managed programme, aims to support the user and increase adherence by presenting the exercises in ways that seniors could identify themselves with, providing behaviour change support and a virtual physiotherapist. As uptake and adherence to exercise is often down to preference, it is important to ascertain whether this application is appealing to older people compared to traditional home exercise booklets. The use of a digital programme may help reach the increasing number of older adults in need of fall prevention.

Little is known about adherence when participants use Otago exercise booklet without the support by home visits or follow-up calls, and longer-term adherence has been sparsely evaluated with e-Health solutions for fall prevention as well. Accordingly, the aim of this participant preference feasibility study was to explore older adults’ participation in a four-month self-managed fall prevention exercise intervention, comparing a digital exercise programme (DP) and a paper booklet (PB). We specifically aimed to describe the participant characteristics and distribution in relation to the self-selected choice of programme, attrition rate, adherence to the programme, experiences and self-reported effects after the intervention, as well as exercise maintenance at one year after study start. The need for individual technical support within the DP group was also studied.

## Method

### Study design

**Fig. 1:**
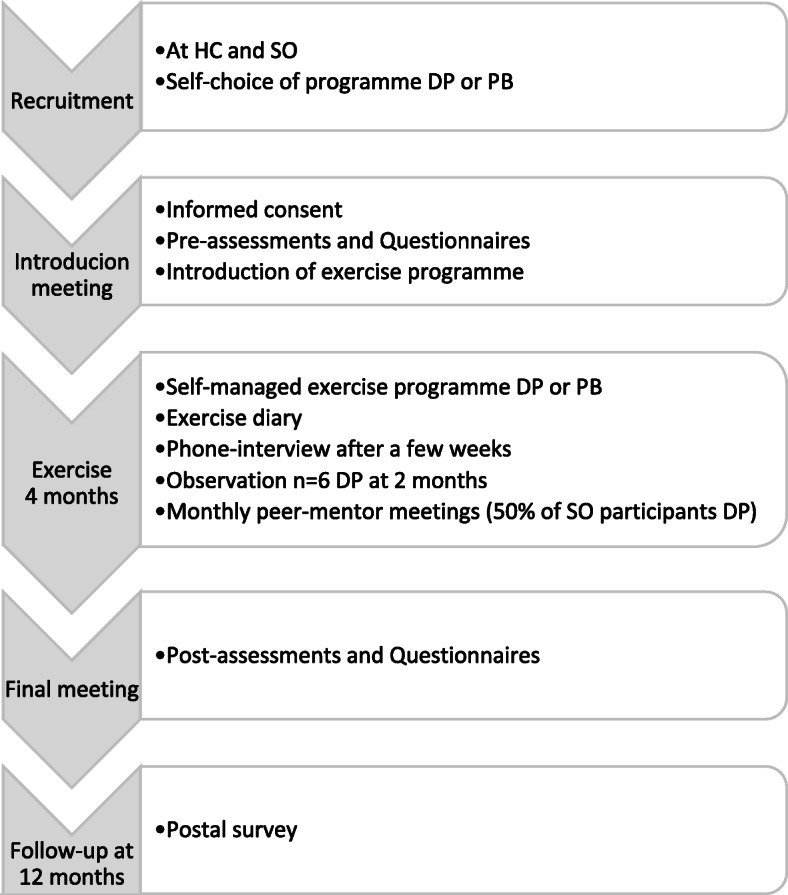
Overview of feasibility study. Key: HC = Health care Centre; SO=Senior citizen Organizations; DP = Digital exercise Programme; PB=Paper Booklet programme

The study was performed to compare two groups using different self-managed exercise programmes (DP or PB). Furthermore, to find out if the new digital programme was feasible for self-management, among seniors accustomed to the use of apps, in a forthcoming large RCT. All participants chose their preferred type of programme, based on their personal preferences and access to technology, no technical devices were provided in this study. After an introduction meeting with pre-assessments the participants exercised independently over four months and self-reported their exercise in an exercise diary. The intervention was completed with a final meeting after four months. Follow-up 12 months after study start was completed using a postal survey. An overview of the study can be seen in Fig. 1. The study is registered in ClinTrial: NCT02916849.

### Participants

Inclusion criteria were: ≥70 years old, living independently, able to rise from a chair and stand without support, experiences of deterioration in balance OR need to be more careful not to lose balance OR have experienced a fall the past year. Exclusion criteria were: doing physical exercise more than 3 h/week, self-reported progressive disease that was likely to influence mobility, and cognitive difficulties. Status of cognitive condition was judged during the screening interview, if the person was able to answer questions satisfactorily, and able to converse about matters regarding the study, they were considered suitable to take part in the study. Participants were recruited at four different senior citizen organisations and at a health care centre. At the senior organizations, information about the project was presented by members of the research team, and contact details of seniors interested in taking part were collected. A research assistant then phoned them for a short interview to screen for suitability to take part. Recruitment at the health care centre was undertaken by a physiotherapist, occupational therapist, nurse, medical doctor or nurse’s aide, who had received an introduction about the programmes sufficient to give information to the potential participants. Participation in the study was voluntary and did not influence the further care of the patient. All participants chose their programme during the first recruitment contact.

### Procedure

Independent of choice of programme, all participants attended an introduction meeting lasting about two hours, including a short presentation about accidental falls and fall prevention, introduction of the exercise programme, and pre-assessments. The respective programme’s main structure, how to select exercises and fill out the exercise diary, and safety aspects during the sessions at home was explained, with opportunity to try some exercises and ask questions. The digital programme group got additional information about the log-in procedure, and how to use parts of the behaviour change support available in the application. Two physiotherapists from the research group led the meetings (LLO, MS), both with experience from the field of fall prevention exercise programmes. Thirteen groups of maximum eight persons met for the introduction meeting. Seven groups from senior citizen organisations had the introduction meeting at the university campus for participants and six were held at the health care centre. The majority of introduction meetings were separate for the DP and PB participants. However, from the health care centre few participants chose the DP, so 2/3 of these meetings were mixed, but the introduction of the actual exercise programme (DP and PB) was kept separate.

Scheduled interaction with the participants during the study was limited as the study focused on self-management of the programmes. A phone interview with all participants was done a few weeks after study start to identify any problems with the programme at an early stage. A help-line phone number was provided in case of encountering any problems while using the programme during the intervention. In order to monitor technical support for the digital program a record of contacts from DP users was kept. Observations by a physiotherapist and a human computer interaction engineer (LM, RJ) were performed with six participants using the DP in their home after approximately eight weeks. A monthly peer-mentor group meeting was held with half of the participants in DP group (recruited at senior citizen organisations), this was also by self-selected choice. These meetings were led by two seniors with a mentor role, together with one of the researchers (MS). Three different topics were discussed, one at each meeting: (1) Initial experiences, (2) Motivation for exercise, and (3) Establishment of lasting exercise routines. The researcher’s role at these meetings did not aim to give extra technical support.

Eight final meetings were held at the university campus and another three at the health care centre. Also participants that had withdrawn from the intervention, by notifying that they stopped exercising, were invited to attend the final meeting to give feedback on the programme.

### Exercise programmes

Both programmes were based on exercises from the Otago Exercise Programme [10] but to provide a variety of exercises at diverse levels, the DP was enriched with both easier and more challenging exercises mainly inspired by the Falls Management Exercise Programme (FaME) [22]. The application for the DP was developed in co-creation with older adults taking their needs and preferences into account. Thus, the exercises are instructed in short video clips imaging older persons doing the exercises, and the user-interface is clean and uncomplicated [21]. The Additional File 1 provides an example of the application’s interface.

In the DP (Safe Step v1 web-based or mobile application) the user builds his or her own exercise programme by selecting one exercise from each of ten predetermined groups of exercises to improve strength, balance and gait/step ability. Each exercise group had several variants of exercises with different levels of difficulty provided by video clips with verbal instructions. The application also included behaviour change support with written motivational feedback from a virtual physiotherapist (computer generated pre-written messages, delivered according to the participants’ reported exercise), exercise planning and possibility to review the exercise diary, as well as examples on how to integrate exercises into daily activities and practice outdoors.

The PB contained the Otago exercises with drawings and written instructions. In order to help the participants build their programme, the exercises were divided in two sections with strength or balance exercises. Each section was further arranged into three different levels of difficulty with was a modification from the Otago Home Exercise Programme Booklet. Participants were instructed to select five exercises from each section to build a programme of ten exercises. Additional exercises for warm-up and stretching included in the booklet were not considered part of the programme’s ten exercises.

In the Supplementary Table 1, Template for Intervention Description and Replication (TIDieR) checklist, a more detailed description of the interventions can be found, Additional File 2.

### Exercise self-management

Participants composed their own programme and exercised independently throughout the four months intervention, directed by material in the programmes and information given at the introduction meeting. Independent of which programme, all participants were asked to choose exercises that they experienced challenging but not too difficult to perform. For balance exercises this meant feeling unstable but without losing balance, and for strength exercises feeling a strain in the muscle but still able to complete the suggested number of repetitions. They were also advised to select new exercises to progress when an exercise became too easy, or to modify if they felt that the exercise they chose became too challenging. The recommendations were to exercise 30 min at least three times per week, according to instructions in the Otago Exercise Programme [10].

### Data collection

Baseline information to describe participants was collected at the introduction meeting with a study specific questionnaire about: age, sex, living condition, education level, fall history, use of walking-aids, self-reported health, and access to technology devices. Activity level was measured with the Saltin-Grimby Physical Activity Level Scale (SGPALS) that also assimilates household activities [23]. Assessment of balance and functional strength was completed using the Short Physical Performance Battery (SPPB) with a maximum score of 12 for the best performance [24], assessed by a physiotherapist blinded to group allocation. Self-rated balance confidence was measured using a translation of the Activities-specific Balance Confidence Scale (ABC), rating from 0 to 100% for 16 activities [25] and a higher score means better confidence. Attitudes to Falls Related Interventions (AFRIS) was determined by a form with six translated statements about the attitude to the programme, to grade if agreed or not on a scale 1–7 to each statement [26], a higher score means a more positive attitude.

Exercise diaries were filled out by the participants over the four months intervention. The exercise diary for the DP allowed self-reporting of: date, which of the predetermined exercises were done and time spent on the practice. The digital diary allowed self-report of exercise once per day, information was stored in a database, from which researchers received data electronically on a monthly basis. The exercise diary for the PB group consisted of a monthly paper sheet, with rows for daily exercise reports, containing the same information as in the DP diary. It was returned in pre-paid envelopes at the end of each month. All diaries were reviewed monthly by the first author (LM), and if there was no data or the data was uncertain the participant was contacted by phone.

A questionnaire developed for this study was answered by the participants at the final meeting. The questionnaire dealt with their experience of using the programme and perceived effects. It had three parts: (1) eleven statements where participants were asked to answer on a Likert type scale from 1 = strongly disagree to 5 = strongly agree (as example “I’m satisfied with the programme I used” or “I notice improved strength in my legs”), (2) two multi-answer questions about positive and negative effects, and (3) further questions about any falls while performing the exercises, if they would recommend the programme to others and if they were going to continue with the programme. If participants did not attend the final meeting the questionnaire was sent out by mail with a pre-paid envelope as their opinions were considered important. Participants that withdrew from the intervention were presented with the option to take part in this questionnaire.

Finally, 12 months after study start a short survey was sent out with a pre-paid envelope to the participants that completed the study and took part in the final meeting (*n* = 45). The aim was to investigate if they continued with the programme, or if not, the reasons why and if they planned to restart.

### Analysis

Differences between groups (based on choice of programme) for baseline characteristics were analysed using Chi-square test (Fisher’s exact test if expected count were < 5), Student’s t-test or Mann-Whitney U-test depending on variable. The activity level of the SGPALS was dichotomized into groups of being inactive (level 1–2) or active (level 3–6) using the same method as Äijö et al. [27]. Withdrawal was noted when participants informed that they stopped exercising with the programme and attrition rate was defined as the proportion of participants that withdraw.

For adherence analyses, the first 16 weeks of self-reported exercise were used. Adherence was described according to guidelines by Hawley-Hague et al. [28] for older adults participating in exercise classes. Their recommendation is to report four types of adherence: completion, attendance, duration, and intensity. We considered two of those to be relevant to our self-management exercise programme: completion, and exercise duration. Four subgroups were created to compare adherence for the DP and the PB with the following definitions:

1. Enrolled, everyone that started the intervention.
2. Completed study, all participants that did not explicitly withdraw from the exercise programme, independent of the degree of participation.
3. Exercise completion ≥75% of the weeks, participants that self-reported exercise at least one session per week for 12 of the 16 weeks.
4. Exercise duration ≥75%, participants that self-reported at least 75% of the recommended 90 min of exercise per week (at least in total 1080 min over 16 weeks).

For each participant, the mean number of minutes and sessions exercised per week were calculated for each week, until the participants stopped reporting to allow for short lapses during the intervention.

Many studies report adherence as percentage of the intended number of sessions over the intervention period, independent on how long time is spent within a session. With the purpose to be able to compare our study with others we also reported these numbers for adherence, recommended number of sessions were 48 over 16 weeks. Outcomes for adherence was analysed with Mann-Whitney U-test. All data were analysed using IBM Corp. Released 2016. IBM SPSS Statistics for Macintosh, Version 24.0. Armonk, NY: IBM Corp.

### Ethical considerations

The study was approved by The Regional Ethical Review Board in Umeå (Dnr 2016/106–31). All participants got written and verbal information about the study and gave written informed consent. Concerns about safety to prevent falls during exercising was considered, it was stressed both during the introduction as well as in the information given in both programmes. Exercise was preferably done close to a wall, sturdy furniture or surface for support, and adapted to the participants’ functional level. Material in the programmes was clear and tailored to the age group to ensure good understanding and reduce any possible risks.

## Results

### Participants

In total, 67 participants were enrolled in the study, 43% chose the DP and 57% chose the PB. Overall there were no major significant differences between groups at study start (Table 1). The mean age was 76 and 77 years respectively, the majority were women and only a minority were physically inactive (SGPALS level 1–2). Over 90% had experienced deterioration in balance during recent years and nearly 60% reported at least one fall the past year (fall range 1–6 falls). Few used walking aids: only two participants (both in the PB group) used a rollator for indoor use, for outdoor use it varied from Nordic walking poles to electric wheelchair. The most prevalent medical condition in both groups were heart- and cardiovascular diseases, and these were significantly less common in the DP group. The DP group also had a significant more positive attitude to the programme at start. Access to smartphone or tablet was significantly higher in the DP group.

**Table 1:** Participants’ background data at start of the intervention

	**Digital programme*n*= 29**	**Paper booklet*n*= 38**	***p*-value**
Age, years, mean ± SD	76 ± 5	77 ± 3	0.508
Women, n (%)	18 (62)	30 (79)	0.173
Living alone, n (%)	13 (45)	17 (45)	0.994
Education, n (%)			0.117
Primary	11 (38)	23 (61)	
Secondary	9 (31)	10 (26)	
Tertiary	9 (31)	5 (13)	
Reduced balance last few years, n (%)	26 (90)	36 (95)	0.645^F^
Fall during previous 12 months	17 (59)	22 (58)	0.952
Indoors	6 (21)	4 (11)	
Outdoors	9 (31)	14 (37)	
Both indoors and outdoors	2 (7)	4 (11)	
Able to take a 5 min brisk walk	25 (86)	34 (90)	0.719^F^
Use of walking aids	4 (14)	10 (27)^†^	0.192
Medical conditions, n (%)			
Heart- and cardiovascular conditions	15 (52)	29 (76)	**0.036**
Neurological conditions	3 (10)	3 (8)	1.0^F^
Musculoskeletal conditions	3 (10)	5 (13)	1.0^F^
Endocrinological conditions	6 (21)	7 (18)	0.816
Lung diseases	3 (10)	6 (16)	0.721^F^
Eye conditions	7 (24)	10 (26)	0.839
Osteoporosis	1 (3)	5 (13)	0.224^F^
Dizziness	7 (24)	9 (24)	0.966
Cancer diagnosis^1^	3 (10)	2 (5)	0.645^F^
Other conditions^2^	0	4 (11)	0.127^F^
Access to smartphone/tablet, n (%)	23 (79)	16 (44)^‡^	**0.004**
Access to computer	26 (90)	27 (75)^‡^	0.130
Inactive (1–2) SGPALS^3^, n (%)			
Summer	1 (3)	7 (18)	0.125^F^
Winter	1 (3)	8 (21)	0.067^F^
SPPB^4^, median (Q1–3)	9 (8–10)	10 (8–10)	0.402
ABC^5^, median (Q1–3)	85 (73–92)	83 (69–89)	0.393
AFRIS^6^, median (Q1–3)	38 (37–42)	37 (36–40)	**0.035**

^1^ Cancer types: Gastrointestinal stromal tumour, Breast ca, Malign melanoma, Chronic lymphocytic leukemia, Prostate ca

^2^ Other: Kidney disease, Ulcerative colitis, Varicose veins, Electro hypersensitivity (EHS)

^3^ Saltin Grimby Physical Activity Level Scale, dichotomized into inactive = level 1–2 and active = level 3–6

^4^ Short Physical Performance Battery, max 12 p

^5^ The Activities-specific Balance Confidence Scale, 0–100%

^6^ Attitudes to Falls Related Interventions, 6–42 p

† 1 person missing

‡ 2 persons missing

^F^*P*-value for Fisher’s exact test

A comparison of participants from the two different recruitment strategies showed significant differences for three background variables (data not reported). The health care centre group self-reported more lung conditions (*p* = .022) and being less physically active during summer months (*p* = .048) than participants recruited from the senior citizen organisations. For participants recruited from the senior citizen organisations access to a computer was significantly higher (*p* < .001).

### Attrition

**Fig. 2:**
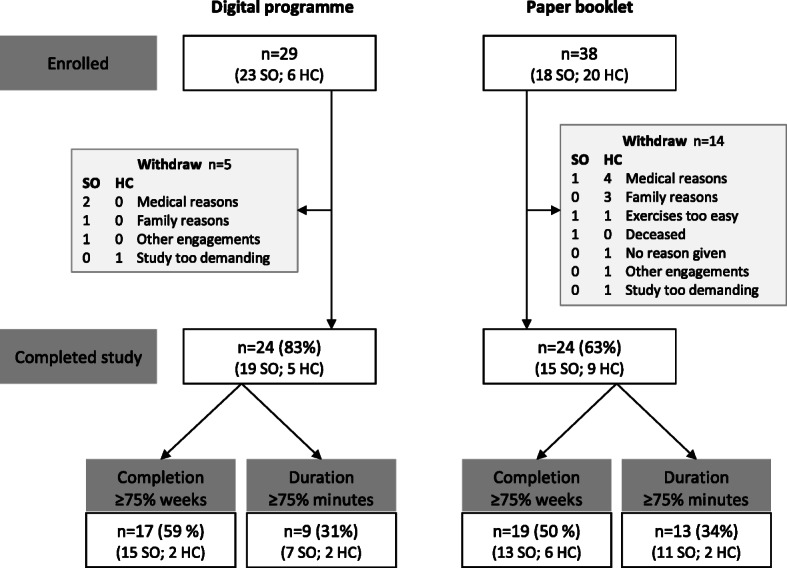
Flow chart of participants’ participation in the study, the distribution of participants from the two recruitment strategies is also shown. Key: SO = Senior citizen Organisations and HC = Health care Centre

Five participants (17%) in the DP group and 14 (37%) in the PB group withdrew explicitly from the intervention, the difference was not statistically significant (*p* = .078) although clinically important. Figure 2 illustrates the flow of participants’ participation in the study. The withdrawals included one after just two days, six during the phone-interview a few weeks into the intervention, and the rest withdrew their participation before or around two months into the intervention. Around 70% of the withdrawals were reported to relate to factors not linked to the intervention programmes: own illness, illness within the family, or other engagements. Two participants stated that the exercises were too easy. One participant died during the period, by causes not related to study participation. Participants not completing the study showed no differences for background characteristics apart from a trend towards a lower education level, which might relate to the slightly higher education level in the DP group. The attrition rate was significant larger in the health care centre group with a 46% withdrawal rate compared to 17% recruited at senior citizen organisations (*p* = .010).

### Adherence

Both intervention programmes had 24 participants completing the study. Among participants using the DP, 17 exercised at least 12 of the 16 weeks (exercise completion ≥75%) and 9 of these reported ≥75% or more of the recommended exercise duration. The corresponding numbers for the PB group were 19 and 13 participants respectively. The DP group and the PB group did not differ significantly in number of participants in these subgroups.

**Fig. 3:**
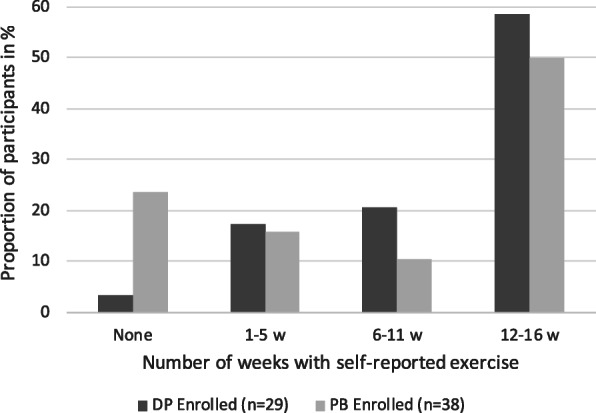
The proportion of enrolled participants for both programmes, reporting exercise completion by number of weeks with at least one self-reported exercise session. Divided in four categories: (a) none, (b) 1–5 weeks, (c) 6–11 weeks, and (d) 12–16 weeks with reported exercise

An illustration summarising weeks of self-reported exercise for both groups is shown in Fig. 3. Of all enrolled participants, 50–59% reported exercise completion ≥75% weeks and a large proportion of PB participants did not report any exercise at all.

**Fig. 4:**
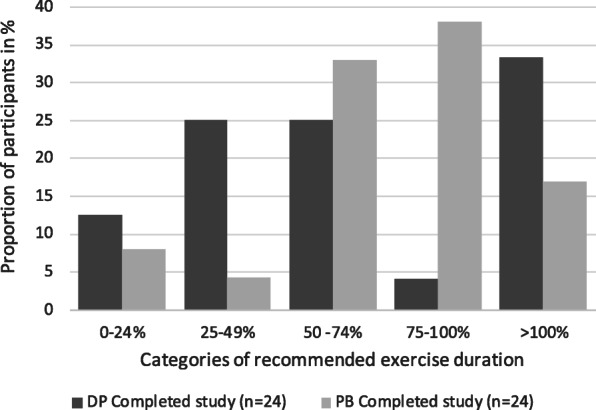
Five categories of self-reported exercise duration (in percent of recommended 90 min per week), shown for those that completed the study (*n* = 24 for each group)

Among those that completed the study there were no significant difference in exercise duration between groups. However, for self-reported total minutes, 55% of the participants in the PB group reached ≥75% exercise duration compared to 37% in the DP group. Conversely, a greater proportion of participants in the DP group exercised more than the recommended duration (>100%) (Fig. 4).

**Fig. 5:**
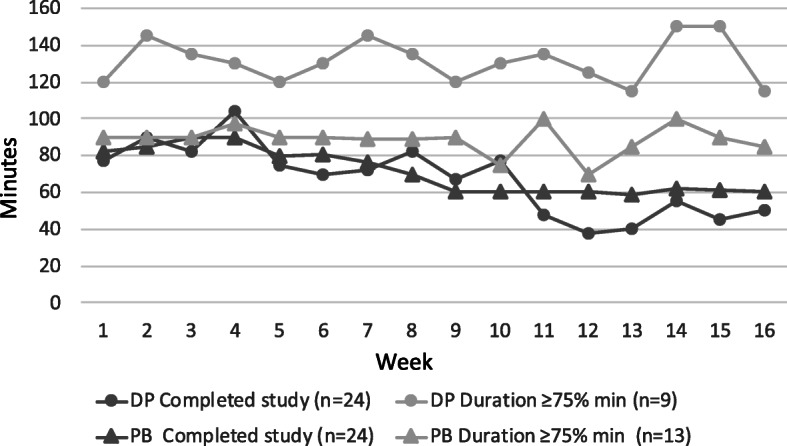
Illustration of median time spent in minutes per week for two subgroups: Completed the study and Exercise duration ≥75%

Reported median exercise time and number of sessions per week is presented in Table 2 with data separate for the four subgroups with respect to adherence. A significant difference between the programmes was only seen in the subgroup completing ≥75% of the recommended exercise duration, with 38 median minutes more per week in the DP group (*p* < .001). In Fig. 5 the change over time can be seen for each programme, between two subgroups (Completed the study and Exercise duration ≥75%). The weekly variation appears larger for the DP group. Around week 12 of the intervention, the Christmas festive season occurred, for the majority of participants which may explain the dip on the graph.

**Table 2:** Self-reported exercise over the intervention period for the four adherence subgroups

	**Digital programme**	**Paper booklet**	***p*-value**
**Enrolled**	n = 29	n = 38	
Total minutes per week	61 (0–110)	65 (0–84)	0.450
Sessions per week	2.3 (1.4–2.9)	2.0 (0.2–2.9)	0.447
Mean adherence of 48 sessions	63%	54%	0.183
**Completed study**	n = 24	n = 24	
Total minutes per week	65 (44–117)	75 (61–88)	0.703
Sessions per week	2.5 (1.8–3.0)	2.7 (2.0–3.0)	0.570
Mean adherence of 48 sessions	74%	80%	0.893
**Exercise completion ≥75% of weeks**	*n* = 17	*n* = 19	
Total minutes per week	86 (58–136)	81 (61–89)	0.375
Sessions per week	2.7 (2.4–3.2)	2.8 (2.0–3.0)	0.557
Mean adherence of 48 sessions	91%	91%	0.505
**Exercise duration ≥75% of recommended minutes**	n = 9	*n* = 13	
Total minutes per week	123 (110–156)	85 (75–94)	**0.001**
Sessions per week	3.1 (2.9–3.8)	2.9 (2.6–3.1)	0.081
Mean adherence of 48 sessions	108%	103%	0.094

Values are expressed as median (Q1–3) for minutes and sessions, and in % for mean adherence

Neither the median number of sessions per week nor adherence as percentage of the recommended number of sessions, did differ significantly between groups for any of the subgroups. Mean adherence of more than 100% reflects participants exercising more than recommended 3 times per week.

A comparison of background data for the subgroup that fulfilled ≥75% of exercise duration (*n* = 22), revealed only that this subgroup was younger with a mean age of 75 (±3), compared to those not reaching ≥75% exercise duration, mean age 78 (±4) (*p* = .029).

Participants in the peer-mentor groups (*n* = 9) reported similar exercise adherence, median 65 (45–95) minutes per week, as participants in the DP group without group meetings (*n* = 15; median 63 (55–148) minutes per week; *p* = .571).

### Technical support

Records of contacts from the DP group showed that support was needed in relation to: self-reporting of exercise *n* = 6, log-in issues n = 6, navigating the programme *n* = 4, hardware issues *n* = 3, Wi-Fi/data plan *n* = 1. The actions to reported problems were: problem was sorted over the phone or by email n = 9, an alert was directed to a person responsible for the server n = 6, or a personal meeting took place n = 3, the problem had resolved and did not need an action to be taken when re-establishing contact *n* = 2. None of the issues were severe to resolve, often the problem occurred due to incorrect handling of the application, or that there was a current server error.

### Survey at four months

The post-assessment questionnaire had a response rate of 93% for participants in the DP group and 71% for the PB group, none of the participants that had withdrawn answered the questionnaire. Table 3, part 1, gives responses for all eleven statements. Both groups were content with their programme, but the DP users reported a significantly higher degree of: being content with the programme and feeling supported by the programme, and also reported perceived improved leg-strength. One statement was boarder significant “difficult to choose exercises at right level” (*p* = .050).

**Table 3:** Results from the post-assessment questionnaire about experiences during the intervention. **Part 1** Statements reflecting degree of agreement to the statement scored from 1 to 5, 1 = strongly disagree, 5 = strongly agree, values are presented as median (Q1–3); **Part 2** Present number of participants experiencing positive and/or negative subjective effects multiple answer possible, values are presented as n (%)

	**Digital program*n*= 27**	**Paper booklet*n*= 27**	***p*-value**
**Part 1**			
Feeling content with the programme	5 (4–5)	4 (3–5)	**.026**
Programme was a support	5 (4–5)	4 (4–5)‡	**.044**
Programme was difficult to use	1 (1–3)	1 (1–3)	.606
Programme contains challenging exercises	4 (3–4)	3.5 (3–5)†	.804
Difficult to choose exercises at right level	1 (1–4)	3 (2–4)†	.050
Worry about safety while practising	1 (1–2)†	1 (1–2)	.696
Practice the same time of the day	3 (2–4)	2.5 (2–3)†	.798
Difficult to find a place to do programme	1 (1–2)	1 (1–3)†	.169
Prefer to work out hard	3 (2–4)	3 (3–4)†	.648
Improved balance	4 (3–5)	4 (3–4)†	.109
Increased leg-strength	4 (3–5)	3 (3–4)†	**.032**
**Part 2**			
Positive effects			
More energy/stamina	13 (48)	7 (26)	.091
Improved mood	3 (11)	7 (26)	.161
Improved well-being	19 (70)	13 (48)	.097
Other	3 (11)	3 (11)	.284
No, no positive effects	0	7 (26)	**.010**^**F**^
Negative effects			
Pain	2 (7)	5 (19)	.224
Dizziness	0	3 (11)	.075
Stress	1 (4)	2 (7)	.552
Tiredness / Fatigue	1 (4)	1 (4)	1.0
Other	1 (4)	2 (7)	.417
No, no negative effects	22 (82)	16 (59)	.074

† 1 person missing

‡ 2 persons missing

^F^ P-value for Fisher’s exact test

Positive effects were reported more than negative effects (Table 3, part 2). The only significant difference between the programmes was that none in the DP group reported that they did not notice any positive effects, compared to 26% in the PB group.

In both groups, 89% would recommend the programme to others, and 82% in the DP group and 70% of PB users planned to continue with the programme after study completion, there were no significant differences between groups.

### Adverse events

One person in the PB group reported two falls during exercise but suffered no injury. For the DP users no falls were reported during exercise.

### Survey at 12 months

The response rate was 98% (24 DP, 20 PB) on the follow-up survey at 12 months after the intervention started. Among DP users 67% exercised regularly, the whole programme or parts of it and significantly more than PB users where the corresponding proportion was 35% (*p* = .036). Among participants from both groups that didn’t continue with the programme or just did it occasionally (*n* = 21) 14% said they would take it up within the month, 43% maybe would take it up again and 10% said that they would not take it up (both from the PB group), and 33% did not answer. The most frequently reported reasons not to continue with the programme was that they had started with other exercises or that they just didn’t do it. Lastly, the 16 DP users that continued with the programme reported that they opened the programme: weekly 19%, monthly 19%, at times 38%, or never 25% (learnt the programme by heart).

## Discussion

This study showed that when participants selected their preferred programme, slightly less participants choose to exercise with the DP, however the attrition rate was higher in the PB group. Despite allocation by participant preference both groups were comparable, no major differences for background characteristics were found. Adherence was generally comparable between the groups, but among frequent users, participants in the DP group reported significantly more minutes of exercise per week in comparison to corresponding PB users. Overall, both groups reported being content with their programme, however DP users reported a higher degree of being content and feeling supported by the programme and they more likely agreed to feeling improved leg-strength. At the 12 months follow-up, 2/3 of participants using the DP continued to exercise regularly compared to 1/3 in the PB group.

### Adherence

The adherence to the programmes was evaluated in the participants own environment as a self-managed exercise intervention. For home exercise programmes many different ways of describing adherence has previously been used. Some studies have reported adherence in minutes or weeks, but adherence in percentage of number of completed sessions is most commonly reported although in various modalities. In our study the completed percentage of recommended number of sessions was equivalent between groups. For all enrolled (subgroup 1); the DP group had 63% adherence, and PB group 54%; for those who completed the study (subgroup 2) the adherence was 74%; and 80% respectively. Adherence in terms of completion of the exercise programme, showed that within both groups half the participants or more reported exercise at least 12 of the 16 weeks (subgroup 3), and one third reached ≥75% of the recommended exercise time (subgroup 4).

Lack of uniformity for reporting adherence makes comparison between studies complex. Moreover, reports of adherence in other self-managed digital fall prevention exercise interventions, are still limited. In our study adherence was measured through self-reports. Several other digital interventions have used the connection time to the application to measure adherence. Considering these dissimilarities, The ActiveLifestyle study [18] indicated similar adherence as in our study: Users of the App with social features had 73% mean adherence of recommended exercise time, the App without social features had 68%, and the Paper programme (control) 54%. In a similar way Dekkeret al [29] reported 68% adherence in a 12 weeks ICT-supported self-management programme, offering an individually tailored fall prevention exercise programme. In comparison, an exergaming intervention iStoppFalls [30], reported 38% adherence as the proportion of participants reaching 1 h exercise per week during their four months intervention.

The PB is based on the Otago Home Exercise Programme, which was originally a programme including regular follow-up, by home visits or telephone calls. In our study the exercise programme was completely self-managed after only one introduction session. Within falls services in the UK, an average of eight group sessions followed by home exercise with a booklet without follow up, is common practice [13]. The Otago Home Exercise Programme has, to our knowledge, not previously been evaluated as a completely self-managed programme but our study indicates that it was a feasible approach with similar adherence levels as for the DP. Studies evaluating the Otago Home Exercise Programme, where home visits were part of the intervention, has shown variable degree of adherence. In a one-year study Liu-Ambrose et al. [31] reported 25% adherence for the recommended exercise three times per week, and 68% for exercise at least once per week. Arkkukangas et al. showed adherence rates of 77% at 12 weeks follow-up [32], and after one year 46% [33]; for a minimum of two exercise sessions per week. In yet another study, the Otago Home Exercise Programme was complemented with DVD support for a six months intervention, where Davis et al. [12] presented 36% adherence, also for minimum two sessions per week.

In a review focused on how different interventions could improve adherence in fall prevention exercise interventions for older adults adherence rates between 27 and 97% was reported [34]. Variation in how adherence was measured limited the interpretation, and a valid objective measurement for both adherence and outcome was called for. Another review targeting exergaming for physical and cognitive effects in older adults, reported 79% mean adherence rate (range 49–96%), however only 1/3 of the studies provided home exercise [35]. Their conclusion was that future research need to evaluate home-based, self-managed interventions and called for robust RCT studies in this field [35]. Both review papers graded, the included studies, as of low evidence quality. Further, both papers address the need to evaluate adherence in a uniformed way, to be able to compare studies [34, 35].

### Attrition and attitude

The attrition rate, the part of participants that withdraw from the study, could be affected by the attitude to an intervention, considering the motivation to realise exercise. Yardley et al. [26] describes the importance of being positive towards the programme to increase participation. The attitude to the programme was assessed with the AFRIS questionnaire at the start of our study and indicated that the participants in the DP group had a more positive attitude to the exercise programme. The significant lower AFRIS score in the PB group was probably a result of a bigger range in scores in this group, which maybe explain the lower attrition rate in this group. Further, the clinically relevant higher attrition rate in the PB group (*p* = .078), could be related to the two recruitment strategies, further discussed as a methodological consideration. Similar attrition rates as ours, of approximately 20%, has been reported in evaluations of other digital exercise interventions over 3–4 months [18, 29, 30]. For the ActiveLifestyle study the control group used a paper programme where attrition rate was 41%, slightly higher than ours [18].

### Exercise maintenance

When following-up with a postal survey after 12 months, as much as 80% of DP and 75% PB participants reported exercising with the programme at times, or even more regularly. In comparison to other fall prevention studies Clemson et al. reported that 57% continued the integrated LiFE programme and 42% the standard programme at 12 months [6] and Iliffe et al. showed that between 40 and 50% of participants in the ActiveLifestyle study were achieving ≥150 min moderate-to-vigorous physical activity per week at 12-month follow-up [11].

The high rate of exercise maintenance after one year, reflected in our survey, may indicate that it was easy to continue doing the exercises when the programme got incorporated in regular activities. Our research group has previously published a qualitative study reporting on participants experiences of partaking in this feasibility study [36] and some of the aspects revealed might explain the high exercise maintenance. Participants from both groups expressed a capability and willingness to manage their exercise independently. The digital programme participants expressed views like: it was easy to choose exercises at an adequate level of difficulty, videos provided useful suggestions how to alter exercises, and videos reduced interpretation of how to perform the exercises. The digital program strengthened the feeling of support, which might create better opportunities for acceptance and adherence in the long term. The conclusion from this qualitative analysis was that the digital programme seems to have supported learning and reflection more than the paper booklet [36].

### Methodological considerations

The reason for the participant preference trial was to evaluate the interest, and use, of a digital programme in a real-life context, and to consolidate the interest for both programmes in this exercise intervention. The patient preference trial is an appealing method to try to improve adherence in interventions [37]. Our study showed better adherence than other, randomised studies [12, 30, 31]. It was positive that even without randomisation the participants’ characteristics were comparable at study start for the two groups. Both groups self-reported relatively good health and would be considered a less frail population. In the PB group more participants indicated to be inactive even though not significantly, so in general we describe both groups as relatively active.

In this study no technical devises were provided to participants, so therefore the significant difference for access to smartphone in the DP group was expected. The intention of this project was to offer this new digital fall prevention programme to persons that have access to and are familiar with technology in order to self-manage this type of programme. This feasibility study was a preparation for a now ongoing large RCT, were the possibility to provide devices will not be possible.

Two different recruitment strategies were used in this study, participants were recruited from a health care centre and through senior citizen organisations. The primary health care service has, in a systematic descriptive review, been considered a good recruitment strategy to identify people for fall preventive exercise interventions [38]. However, we found that fewer participants from the health care centre (primary care) chose the digital programme, which can have many possible reasons. Firstly, health care professionals imparted the information at the health care centre and they maybe had less time to explain the programmes, which might have affected the selection of programme. Secondly, a more detailed description of the new digital programme may be needed to make an informed choice. The preunderstanding of a paper booklet might have favoured the choice of this programme, as choosing the known might be an easier choice. Thirdly, access to a computer was significantly lower among the health care centre recruits, and may also reflect their previous experience of using technology who did not favour the DP. Furthermore, participants from the health care centre were less likely to finish the intervention which might explain parts of the higher attrition rate among PB users. Participants recruited at a health care centre might also have expected some other treatment, rather than self-management through a home exercise programme, which may influence their attitude towards the programme. These two different recruitment strategies could be further investigated, to be able to find an effective way to implement this type of fall preventive initiative.

To evaluate adherence, and compare the actual use of the two programmes, we created four subgroups according to participation in the intervention. Just over half of the participants completed more than 12 of the 16 weeks (75%) of the intervention (subgroup 3), and one third of the participants completed ≥75% duration for reported exercise time (subgroup 4). Different studies have used different cut-off points, we based our (75%), on the review by Hawley-Hague et al. [28] and the ActiveLifestyle study Van het Reve et al. [19]. Using this cut-off point allows for some short illness during the study period, or lapses due to holidays or festive season. However, these recommendations for definitions of adherence were based on participation in exercise classes [28], consequently it could not be followed entirely in our study.

In our study adherence was based on data from exercise diaries, with self-reported time spent performing the exercises and what exercises that were performed. Maybe participants’ aspiration to accomplish the task exactly as recommended, lead to over or under estimation of self-reported time. A study exploring self-reported physical activity compared to accelerometer measured activity, showed an over estimation of the active time when self-reporting [39]. Further, comparing paper and electronic diaries in pain patients, showed a high frequency of bogus entries in both groups, while hording and/or filling in in advance was common for the paper diary as no time constraint existed to enter data. Also a poorer compliance was reported for the paper diary [40]. In our study, the digital exercise diary permitted data entry once per day, to report retrospectively but not in advance, and the paper diary had no limitations. The DP users predominantly reported either a lot more or less than recommended exercise time. Possible explanations for this peculiar u-shaped pattern was gained from participants during the final meeting where some explained that they regularly practiced without reporting it in the application. Others stopped using the application regularly while exercising, as they learned the programme by heart and did not open the application. In that sense a paper diary was easier to access, and such oversights could explain the low numbers of DP users in the span 75–100% of exercise duration. Additionally, the monthly reviews of the DP diaries were not realised in the penultimate month (as the data was not provided to the research assistant), causing a plausible risk that the adherence for the second part of the study in the DP group was affected. Other studies have used the actual connectivity of the app or exergame to monitor adherence [18, 29, 30], but the question rise if an over reporting of adherence may occur, when e.g. every connection to show someone the programme or go back and repeat watching a section, will be reported as exercise. There is a need for more studies to evaluate adherence to new digital fall prevention interventions and consistency in the way adherence is assessed.

## Conclusion

The main goal of this feasibility study was to explore older adults’ participation in a four-month self-managed fall prevention exercise intervention and specifically compare adherence when using a digital exercise programme or a paper booklet. Among participants who completed the study adherence was similar between the digital and paper programme. However, the PB group had a clinically relevant higher attrition rate, and frequent users in the DP group reported more exercise. A lesson learned was that collecting data on adherence is complex and the different ways of self-reporting exercise may have affected the results. With respect to the complexity of adherence, our study showed similar adherence as other published studies of digital exercise fall prevention programmes and even higher than in other studies of paper-based programmes. Overall, participants who completed the study were satisfied with the chosen programme and at 12 months follow-up a greater proportion of participants using the DP continued to exercise regularly. The results from this study shows that both the digital programme and the paper programme have potential to be used as self-managed fall prevention exercise interventions and the digital programme seems to facilitate regular exercise after the intervention period. In a coming RCT study the digital programme will be investigated without any in-person interaction.

## Supplementary information

**Additional file 1.** Example of the application’s interface (Safe Step v1 web-based or mobile application) with translation from Swedish. Please see Additional file 2 for more information about the content in the Safe Step application v1.

**Additional file 2: Supplementary Table 1**. Template for Intervention Description and Replication (TIDieR) checklist for Safe Step feasibility study

## Data Availability

The data used to support the findings of this study are available from the corresponding author on reasonable request.

## Abbreviations

DP: Digital exercise Programme
HC: Health care Centre
PB: Exercise Paper Booklet programme
SGPALS: Saltin-Grimby Physical Activity Level Scale
SO: Senior citizen Organisations

## References

[1] Sherrington C, Fairhall NJ, Wallbank GK, Tiedemann A, Michaleff ZA, Howard K, et al. Exercise for preventing falls in older people living in the community. Cochrane Database Syst Rev. 2019. 10.1002/14651858.CD012424.pub2. 10.1002/14651858.CD012424.pub2 PMC6360922 30703272

[2] Hamed A, Bohm S, Mersmann F, Arampatzis A (2018). Follow-up efficacy of physical exercise interventions on fall incidence and fall risk in healthy older adults: a systematic review and meta-analysis. Sports medicine-open.

[3] Florence CS, Bergen G, Atherly A, Burns E, Stevens J, Drake C (2018). Medical costs of fatal and nonfatal falls in older adults. J Am Geriatr Soc.

[4] Kempen GI, van Haastregt JC, McKee KJ, Delbaere K, Zijlstra GR (2009). Socio-demographic, health-related and psychosocial correlates of fear of falling and avoidance of activity in community-living older persons who avoid activity due to fear of falling. BMC Public Health.

[5] Kendrick D, Kumar A, Carpenter H (2014). Zijlstra GR.

[6] Clemson L, Singh MAF, Bundy A, Cumming RG, Manollaras K, O’Loughlin P (2012). Integration of balance and strength training into daily life activity to reduce rate of falls in older people (the LiFE study): randomised parallel trial. Bmj..

[7] Centers for Disease Control and Prevention, U.S. Department of Health & Human Services: CDC Compendium of Effective Fall Interventions: What Works for Community-Dwelling Older Adults, 3rd Edition. https://www.cdc.gov/homeandrecreationalsafety/falls/compendium.html. Accessed 28 Nov 2018.

[8] Public Health England, GOV.UK: Falls and fractures consensus statement: resource pack. https://www.gov.uk/government/publications/falls-and-fractures-consensus-statement. Accessed 28 Nov 2018.

[9] Department of Health, Queensland Government: Otago Exercise Programme - Qld Stay On Your Feet. https://www.health.qld.gov.au/stayonyourfeet/for-professionals/otago. Accessed 28 Nov 2018.

[10] Gardner MM, Buchner DM, Robertson MC, Campbell AJ (2001). Practical implementation of an exercise-based falls prevention programme. Age Ageing.

[11] Iliffe S, Kendrick D, Morris R, Griffin M, Haworth D, Carpenter H (2015). Promoting physical activity in older people in general practice: ProAct65+ cluster randomised controlled trial. Br J Gen Pract.

[12] Davis JC, Hsu CL, Cheung W, Brasher PM, Li LC, Khan KM (2016). Can the Otago falls prevention program be delivered by video?. A feasibility study BMJ open sport & exercise medicine.

[13] Royal College of Physicians, UK. Older people’s experience of therapeutic exercise as part of a falls prevention service: https://www.rcplondon.ac.uk/projects/outputs/older-peoples-experience-therapeutic-exercise-part-falls-prevention-service. Accessed 31 Oct 2018.

[14] Robinson L, Dawson P, Newton J. Promoting adherence with exercise-based falls prevention programmes. In: Vincent ML Moreau, TM (eds), editor. Accidental Falls: Causes, Prevention and Intervention. New York: Nova Science Publishers,; 2008:283–98.

[15] Sherrington C, Michaleff ZA, Fairhall N, Paul SS, Tiedemann A, Whitney J (2017). Exercise to prevent falls in older adults: an updated systematic review and meta-analysis. Brit J Sport Med.

[16] Lesinski M, Hortobágyi T, Muehlbauer T, Gollhofer A, Granacher U (2015). Effects of balance training on balance performance in healthy older adults: a systematic review and meta-analysis. Sports Med.

[17] Valenzuela T, Okubo Y, Woodbury A, Lord SR, Delbaere K (2018). Adherence to technology-based exercise programs in older adults: a systematic review. J Geriatr Phys Ther.

[18] Silveira P, Van De Langenberg R, Van Het Reve E, Daniel F, Casati F, De Bruin ED (2013). Tablet-based strength-balance training to motivate and improve adherence to exercise in independently living older people: a phase II preclinical exploratory trial. J Med Internet Res.

[19] van Het Reve E, Silveira P, Daniel F, Casati F, De Bruin ED (2014). Tablet-based strength-balance training to motivate and improve adherence to exercise in independently living older people: part 2 of a phase II preclinical exploratory trial. J Med Internet Res.

[20] Delbaere K, Valenzuela T, Woodbury A, Davies T, Yeong J, Steffens D (2015). Evaluating the effectiveness of a home-based exercise programme delivered through a tablet computer for preventing falls in older community-dwelling people over 2 years: study protocol for the standing tall randomised controlled trial. BMJ Open.

[21] Sandlund M, Lindgren H, Pohl P, Melander-Wikman A, Bergvall-Kåreborn B, Lundin-Olsson L. Towards a mobile exercise application to prevent falls: a participatory design process. In: 10th Intl Conf. Disability, Virtual Reality & Associated Technologies. Gothenburg, Sweden; 2014.

[22] Skelton DA, Dinan SM (1999). Exercise for falls management: rationale for an exercise programme aimed at reducing postural instability. Physiotherapy theory and practice.

[23] Grimby G, Frändin K (2018). On the use of a six-level scale for physical activity. Scand J Med Sci Sports.

[24] Guralnik JM, Simonsick EM, Ferrucci L, Glynn RJ, Berkman LF, Blazer DG (1994). A short physical performance battery assessing lower extremity function: association with self-reported disability and prediction of mortality and nursing home admission. J Gerontol.

[25] Powell LE, Myers AM (1995). The activities-specific balance confidence (ABC) scale. J Gerontol Ser A Biol Med Sci.

[26] Yardley L, Donovan-Hall M, Francis K, Todd C (2007). Attitudes and beliefs that predict older people’s intention to undertake strength and balance training. J Gerontol Ser B Psychol Sci Soc Sci.

[27] Äijö M, Kauppinen M, Kujala UM, Parkatti T (2016). Physical activity, fitness, and all-cause mortality: an 18-year follow-up among old people. J Sport Health Sci.

[28] Hawley-Hague H, Horne M, Skelton DA, Todd C (2016). Review of how we should define (and measure) adherence in studies examining older adults’ participation in exercise classes. BMJ Open.

[29] Dekker-van Weering M, Jansen-Kosterink S, Frazer S, Vollenbroek-Hutten M. User Experience, Actual Use, and Effectiveness of an Information Communication Technology-Supported Home Exercise Program for Pre-Frail Older Adults. Front Med (Lausanne). 2017;4. doi:10.3389/fmed.2017.00208. 10.3389/fmed.2017.00208 PMC5715376 29250523

[30] Gschwind YJ, Eichberg S, Ejupi A, de Rosario H, Kroll M, Marston HR, et al. ICT-based system to predict and prevent falls (iStoppFalls): results from an international multicenter randomized controlled trial. Eur Rev Aging Phys Act. 2015;12:10. 10.1186/s11556-015-0155-6 PMC4748323 26865874

[31] Liu-Ambrose T, Donaldson MG, Ahamed Y, Graf P, Cook WL, Close J (2008). Otago home-based strength and balance retraining improves executive functioning in older fallers: a randomized controlled trial. J Am Geriatr Soc.

[32] Arkkukangas M, Söderlund A, Eriksson S, Johansson A-C (2019). Fall preventive exercise with or without behavior change support for community-dwelling older adults: a randomized controlled trial with short-term follow-up. J Geriatr Phys Ther.

[33] Arkkukangas M, Söderlund A, Eriksson S, Johansson A-C (2018). One-year adherence to the Otago exercise program with or without motivational interviewing in community-dwelling older adults. J Aging Phys Act.

[34] Hughes KJ, Salmon N, Galvin R, Casey B, Clifford AM (2019). Interventions to improve adherence to exercise therapy for falls prevention in community-dwelling older adults: systematic review and meta-analysis. Age Ageing.

[35] Howes SC, Charles DK, Marley J, Pedlow K, McDonough SM (2017). Gaming for health: systematic review and meta-analysis of the physical and cognitive effects of active computer gaming in older adults. Phys Ther.

[36] Pettersson B, Wiklund M, Janols R, Lindgren H, Lundin-Olsson L, Skelton DA (2019). ‘Managing pieces of a personal puzzle’ — older people’s experiences of self-management falls prevention exercise guided by a digital program or a booklet. BMC Geriatr.

[37] Brewin CR, Bradley C (1989). Patient preferences and randomised clinical trials. BMJ: British Medical Journal.

[38] Shier V, Trieu E, Ganz DA (2016). Implementing exercise programs to prevent falls: systematic descriptive review. Injury epidemiology.

[39] Dyrstad SM, Hansen BH, Holme IM, Anderssen SA (2014). Comparison of self-reported versus accelerometer-measured physical activity. Med Sci Sports Exerc.

[40] Stone AA, Shiffman S, Schwartz JE, Broderick JE, Hufford MR (2003). Patient compliance with paper and electronic diaries. Control Clin Trials.

