# Supplementary material for: Older adults’ preferences for, adherence to and experiences of two self-management falls prevention home exercise programmes: a comparison between a digital programme and a paper booklet
Source: BMC Geriatr. 2020 Jun 15;20:209. doi: 10.1186/s12877-020-01592-x (PMC7294667; doi:10.1186/s12877-020-01592-x)
Supplement: Supplementary file 1 — Additional file 1. Example of the application’s interface (Safe Step v1 web-based or mobile application) with translation from Swedish. Please see Additional file 2 for more information about the content in the Safe Step application v1. [file 12877_2020_1592_MOESM1_ESM.pdf]

Additional File 1.

Example of the application’s interface (Safe Step v1 web-based or mobile application) with translation from Swedish. Please see Additional File 2 for more information about the content in the Safe Step application v1.

Safe Step Home screen

My Exercises

Exercise Diary

Integrate exercises into everyday activities

More about Safe Step

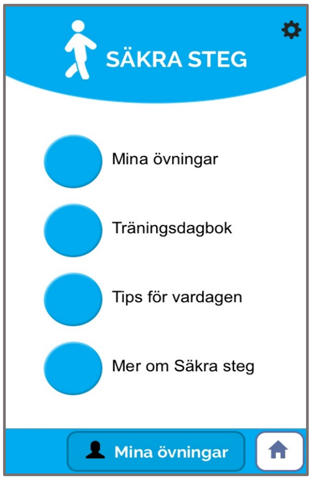

My Exercises screen

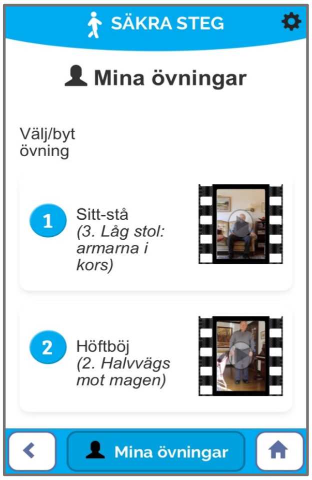

My Exercises

Choose/change exercise

Sit to stand
(3. Low chair: arms crossed over chest)

Hip flexion
(2. Half-way to the belly)

The home screen with four options:

- Go to the exercise programme (My Exercises)
- Access the exercise diary (Exercise Diary)
- Get hints about how to integrate exercises into everyday activities or outdoor (Integrate exercises into everyday activities)
- Get more information about the application (More about Safe Step)

The screen for My Exercises, with example of Sit to stand and Hip flexion exercises.

- To choose/change exercise click on the circle with a number (1-10 for the ten exercises) to show the list of selectable exercises.
- The video clip with exercise instructions starts and expands when clicking on the movie icon.
