# Supplementary material for: Older adults’ preferences for, adherence to and experiences of two self-management falls prevention home exercise programmes: a comparison between a digital programme and a paper booklet
Source: BMC Geriatr. 2020 Jun 15;20:209. doi: 10.1186/s12877-020-01592-x (PMC7294667; doi:10.1186/s12877-020-01592-x)
Supplement: Supplementary file 2 — Additional file 2: Supplementary Table 1. Template for Intervention Description and Replication (TIDieR) checklist for Safe Step feasibility study [file 12877_2020_1592_MOESM2_ESM.pdf]

Additional File 2. Supplementary Table 1. Template for Intervention Description and Replication (TIDieR) checklist for Safe Step feasibility study

Item no./Name	Description	
1. Brief name	Safe Step<br>A feasibility study of fall preventive exercise interventions delivered as a digital exercise programme or a paper booklet programme. The digital programme was Safe Step (v1), a web-based or mobile application and the paper booklet was a modified version of the Otago Exercise Programme.	
2. Why	A self-managed digital exercise program has been developed for community-living older people in order to potentially increase access to and reduce costs of fall prevention exercise. Before evaluation in a larger randomized controlled trial this feasibility study was conducted to evaluate the digital programme in comparison to a paper-based programme with respect to attrition, adherence, and self-reported experiences and effects.	
3. What: Materials	Both programmes provide a repository of evidence based exercises and were developed with inspiration from exercises in the Otago Exercise Programme (OEP), additionally the digital programme have exercises inspired from Falls Management Exercise Programme (FaME). Participants were instructed to build a complete programme of 10 different exercises.	
	**Safe Step digital programme**<br>• Video clips with verbal instructions.<br>• A repository of exercises organized into 10 exercise groups: 5 to increase lower-limb muscle strength, 2 to improve balance, and 3 gait- and step exercises. Each group contained several exercises with varied levels of difficulty.<br>• Reasons for doing the exercise and how to adapt the exercise (for e.g. knee pain) were provided for each exercise group.<br>• Examples of exercises integrated into everyday activities and tips on how to do exercises outdoors.<br>• Behavior change support including: motivational feedback with written messages from virtual physio-therapist, weekly activity planning, and monitoring progress (i.e. previous reported activities and frequency of exercise).<br>• Safety advice was provided in each exercise video and also for every exercise group. In addition, general safety information for everyday life was provided.<br>• Integrated self-reported digital exercise diary. Data saved on a secure server and reports monthly to the research group from an administrator.	**Paper booklet programme**<br>• Drawings with written instructions.<br>• A repository of exercises organized into two sections of strength- and balance exercises. Each section was divided into three different levels of difficulty.<br>• The booklet had additional exercises for warm-up and stretching.<br>• Adaptation of exercises and reasons for doing the exercises were not provided.<br>• No integrated exercise practice was provided.<br>• No feedback or weekly planning tool was provided.<br>• The first page in the booklet presented general safety instructions for doing the exercise programme.<br>• Self-reported paper-based exercise diary sent monthly to the research group.

4. What: Procedures	Participants self-selected their preferred intervention programme before study start. An introduction meeting included a short physical assessment, completion of questionnaires, and a short introduction of the exercise programme. At the end of the intervention a similar final meeting with post-assessments was held.<br>A contact number to the project group was supplied if problems with the programme arose during the intervention.
5. Who provided	The intervention was self-managed and the participants chose the exercises and performed the programme independently over 4 months.
6. How	The individual programme was commenced after an introduction meeting. A short introduction of the programme was held in small groups of max 8 participants. The instructions were to build a programme with 10 different exercises from the repository. Participants were recommended to try the different exercises in each group, starting with the easiest and then choose one that was challenging but not too difficult. The digital programme was delivered via computer, mobile phone or tablet and the paper programme was delivered through a booklet.<br><br>**Safe Step digital programme**<br>• In video clips, the verbal instructions guided participants to start with 2 sets of 5 repetitions and continue to 10 reps before trying a more challenging exercise.<br><br>**Paper booklet programme**<br>• Instruction page guided participants to start with 2 sets of 5 repetitions and continue to 10 reps.
7. Where	The participants decided themselves were to exercise, most probably in their own home or near environment. The introduction and final meetings were held at the recruiting health care centre or at the university campus.
8. When and how much	The recommendation was to exercise with the programme for 30 min, at least 3 times per week over 4 months, with instruction to progress the level of difficulty over the period according to their ability.
9. Tailoring	The participants were instructed to self-tailor the programme by choosing exercises that were challenging but not too difficult to perform, meaning exercising without losing balance or not being able to complete the number of repetitions. The individual programme could be progressed and tailored at any time throughout the 4-month intervention.
10. Modifications	N/A
11. How well: Planned	Adherence was self-reported by the participants in an exercise diary by recording exercises and time spent for each session and followed up monthly by the research team. In case of delay in exercise diary entry at the end of the month a phone call was made as a reminder.
11. How well: Actual	A drawback with participants individual exercise diaries was irregular data reporting from some participants during parts of the study. The attrition rate was 17% for Safe Step and 37% for paper booklet.
